# Projected costs of single-payer healthcare financing in the United States: A systematic review of economic analyses

**DOI:** 10.1371/journal.pmed.1003013

**Published:** 2020-01-15

**Authors:** Christopher Cai, Jackson Runte, Isabel Ostrer, Kacey Berry, Ninez Ponce, Michael Rodriguez, Stefano Bertozzi, Justin S. White, James G. Kahn

**Affiliations:** 1 UCSF School of Medicine, University of California, San Francisco, San Francisco, California, United States of America; 2 UCLA Fielding School of Public Health, University of California, Los Angeles, Los Angeles, California, United States of America; 3 David Geffen School of Medicine at UCLA, University of California, Los Angeles, Los Angeles, California, United States of America; 4 School of Public Health, University of California Berkeley, Berkeley, California, United States of America; Massachusetts General Hospital, UNITED STATES

## Abstract

**Background:**

The United States is the only high-income nation without universal, government-funded or -mandated health insurance employing a unified payment system. The US multi-payer system leaves residents uninsured or underinsured, despite overall healthcare costs far above other nations. Single-payer (often referred to as Medicare for All), a proposed policy solution since 1990, is receiving renewed press attention and popular support. Our review seeks to assess the projected cost impact of a single-payer approach.

**Methods and findings:**

We conducted our literature search between June 1 and December 31, 2018, without start date restriction for included studies. We surveyed an expert panel and searched PubMed, Google, Google Scholar, and preexisting lists for formal economic studies of the projected costs of single-payer plans for the US or for individual states. Reviewer pairs extracted data on methods and findings using a template. We quantified changes in total costs standardized to percentage of contemporaneous healthcare spending. Additionally, we quantified cost changes by subtype, such as costs due to increased healthcare utilization and savings due to simplified payment administration, lower drug costs, and other factors. We further examined how modeling assumptions affected results. Our search yielded economic analyses of the cost of 22 single-payer plans over the past 30 years. Exclusions were due to inadequate technical data or assuming a substantial ongoing role for private insurers. We found that 19 (86%) of the analyses predicted net savings (median net result was a savings of 3.46% of total costs) in the first year of program operation and 20 (91%) predicted savings over several years; anticipated growth rates would result in long-term net savings for all plans. The largest source of savings was simplified payment administration (median 8.8%), and the best predictors of net savings were the magnitude of utilization increase, and savings on administration and drug costs (*R*^2^ of 0.035, 0.43, and 0.62, respectively). Only drug cost savings remained significant in multivariate analysis. Included studies were heterogeneous in methods, which precluded us from conducting a formal meta-analysis.

**Conclusions:**

In this systematic review, we found a high degree of analytic consensus for the fiscal feasibility of a single-payer approach in the US. Actual costs will depend on plan features and implementation. Future research should refine estimates of the effects of coverage expansion on utilization, evaluate provider administrative costs in varied existing single-payer systems, analyze implementation options, and evaluate US-based single-payer programs, as available.

## Abstract 2

In a systematic review, Christopher Cai and colleagues investigate the projected cost impact associated with a single-payer approach to health insurance in the United States.

## Author summary

**Why was this study done?:**

- As the US healthcare debate continues, there is growing interest in “single-payer” also known as “Medicare for All.” Single-payer uses a simplified public funding approach to provide everyone with high-quality health insurance.
- Public support for provision of universal health coverage through a plan like Medicare for All is as high as 70%, but falls when costs are emphasized.
- Economic models help assess the financial viability of single-payer. Yet, models vary widely in their assumptions and methods, and can be hard to compare.

**What did the researchers do and find?:**

- We found and compared cost analyses of 22 single-payer plans for the US or individual states.
- Nineteen (86%) of the analyses estimated that health expenditures would fall in the first year, and all suggested the potential for long-term cost savings.
- The largest savings were predicted to come from simplified billing and lower drug costs.
- Studies funded by organizations across the political spectrum estimated savings for single-payer.

**What do these findings mean?:**

- There is near-consensus in these analyses that single-payer would reduce health expenditures while providing high-quality insurance to all US residents.
- To achieve net savings, single-payer plans rely on simplified billing and negotiated drug price reductions, as well as global budgets to control spending growth over time.
- Replacing private insurers with a public system is expected to achieve lower net healthcare costs.

## Introduction

Nine years after passage of the Affordable Care Act, 10.4% (27.9 million) of the nonelderly US population remains uninsured [1]. Lack of insurance is associated with worse health outcomes, including death [2], due to decreased access to healthcare and preventive services [3–5]. Underinsurance, defined as cost sharing that represents significant financial barriers to care or risk of catastrophic medical expenditures, is rising and is associated with a 25% or greater likelihood of omitted or delayed care [6,7]. Low-income adults with public insurance have improved access and quality of care compared to uninsured adults [8].

Meanwhile, healthcare costs continue to rise, approaching one-fifth of the economy. In 2018, national health expenditures reached $3.6 trillion, equivalent to 17.7% of GDP [9]. Government funding, including public programs, private insurance for government employees, and tax subsidies for private insurance, represented 64% of national health expenditures in 2013, or 11% of GDP, more than total health expenditures in almost any other nation [10]. Higher costs in the US are due primarily to higher prices and administrative inefficiency, not higher utilization [11–13].

An oft-proposed alternative to the contemporary multi-payer system is single-payer, also referred to as Medicare for All. Key elements of single-payer include unified government or quasi-government financing, universal coverage with a single comprehensive benefit package, elimination of private insurers, and universal negotiation of provider reimbursement and drug prices. Single-payer as it has been proposed in the US has no or minimal cost sharing. Polled support for single-payer is near an all-time high, as high as two-thirds of Americans [14] and 55% of physicians [15]. Two-thirds of Americans support providing universal health coverage through a national plan like Medicare for All as an extremely high priority for the incoming Congress [16]. However, support varies substantially according to how single-payer is described [17]. As of November 2019, there are 2 “Medicare for All Act of 2019” legislative proposals in the US Congress: Senate Bill 1129 and House of Representatives Bill 1384.

Economic analyses are crucial for formally estimating the net cost of single-payer proposals. These models estimate how potential added costs of single-payer, due to increased utilization of services, compare with the savings induced by simplified payment administration, lower drug prices, and other factors. Such economic projections can shape plan design, contribute to policy discourse, and affect the viability of legislation. As single-payer proposals gain legislative traction, the importance of economic models rises.

However, these analyses are complex and heterogeneous, making generalizations difficult. Findings vary across studies, from large “net savings” to “net costs,” as do modeling assumptions, such as the extent of administrative savings and presence or absence of drug price negotiations. The diversity of findings contributes to political spin and fuels popular uncertainty over the anticipated costs of a single-payer healthcare system. For example, a 2018 study by Pollin et al. (Political Economy Research Institute) estimated that a national Medicare for All system would save $313 million in the first year of implementation, while a 2018 study by Blahous (Mercatus Center) found that the same system would save $93 million in the first year, and a 2016 report from Holahan et al. (Urban Institute) suggested that a modified form of this proposal, e.g., relying on private insurers, would increase costs [18–20]. Variation in single-payer proposals and analytic approaches likely explains many of the differences in outcomes across studies, but no comparative review has been undertaken, to our knowledge.

The goal of this study is to systematically review economic analyses of the cost of single-payer proposals in the US (both national and state level), summarize results in a logical but accessible manner, examine the association of findings with plan features and with analytic methods, and, finally, examine the empirical evidence regarding key study assumptions.

## Methods

### Overview

We specified in advance that we would extract and quantitatively compare increased costs due to utilization rises and savings due to administrative simplification, drug savings, and other factors. We searched for studies by examining existing lists, querying experts, and searching online. Ethics approval was not deemed to be necessary since all data were publicly available. All data are available in the original studies, which are listed in S1 Appendix. We included studies that examined insurance plans with essential single-payer features and that provided adequate technical detail on inputs and results. For these studies, we extracted information about plan features, analytic assumptions, and findings (costs due to higher utilization, savings of all types, and net costs; see Table A in S1 Appendix for definitions of terms). We expressed all estimates as a percentage of contemporaneous healthcare spending, to facilitate comparison across settings and time periods. We summarized study methods and findings graphically and analyzed associations between studies and spending estimates.

### Search

We adopted a broad search strategy, reflecting our initial assessment (subsequently confirmed) that economic models of the cost of single-payer plans are not published in academic journals. We conducted all components of our search from June 1 to December 31 of 2018.

**Fig 1:**
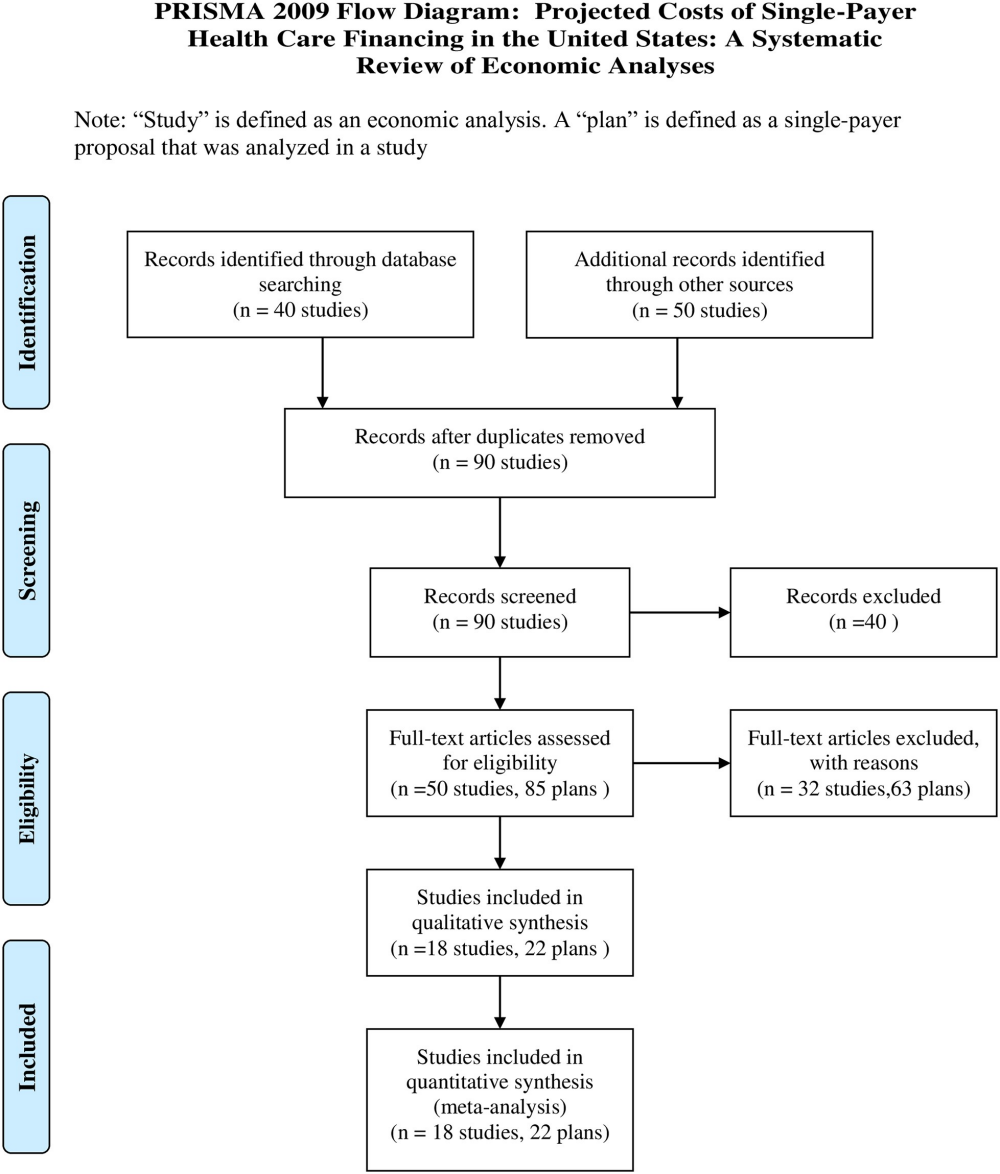
PRISMA flow diagram.

We searched in PubMed, Google Scholar, and Google, using combinations of (“Single-payer” OR “single-payer”) AND (“cost” OR “model” OR “economic” OR “cost-benefit”). We limited our Google search to 10 pages of results. We consulted existing lists maintained by Physicians for a National Health Program and Healthcare-NOW [21,22]. We asked a convenience sample of 10 single-payer experts. We also searched the websites of leading advocacy and industry-sponsored groups in favor of single-payer reform (Physicians for a National Health Plan and Healthcare-NOW) and in opposition to single-payer reform (Partnership for America’s Health Care Future). Additional search details are provided in Table B in S1 Appendix. A PRISMA flow diagram is provided in Fig 1. A PRISMA checklist can be found in Table G in S1 Appendix.

### Inclusion and exclusion

“Single-payer” has a wide range of definitions, both in the US and internationally. We chose inclusion and exclusion criteria that were most consistent with single-payer plans that have been proposed in the US. For example, while some single-payer plans internationally have included private intermediaries within a unified payment system, US proposals have omitted a role for private insurers. Thus, we use private intermediaries as an exclusion criterion. Notably, recent healthcare proposals such as “Medicare Extra for All” would not meet our inclusion criteria [23].

Study inclusion required appropriateness of both the plan and the analysis. Specific inclusion criteria for the plan were that (1) all legal residents are permanently covered for a standard comprehensive set of medically appropriate outpatient and inpatient medical services under one payer and (2) the payer is a not-for-profit government or quasi-government agency. Other central single-payer features, such as providers being entirely in or out, uniform payments with no balance billing, and use of a drug formulary, are often unspecified and thus were assumed present (and thus not a basis for exclusion) unless explicitly omitted. Some plans include undocumented immigrants, and some exclude them. Exclusion criteria were (1) use of large cost-sharing measures such as deductibles (some US single-payer plans include small copays, e.g., $5–$10 for an outpatient visit, which was not considered grounds for exclusion) and (2) an explicit role for non-uniform payment levels (i.e., payments differing by patient), balance billing, multiple payment systems, multiple drug formularies, or private insurers or intermediaries. Importantly, we applied these criteria to the modeled plan, so models incorporating any of these features when analyzing an otherwise qualifying single-payer plan would be excluded. These excluded studies are listed in Table C in S1 Appendix. Finally, we excluded 12 plans from 11 studies that met inclusion criteria but were redundant to newer studies of similar single-payer plans by the same analysis teams already included (Table D in S1 Appendix). Net savings from these excluded studies were similar to those from the included studies (Table E in S1 Appendix).

For the analysis, all studies were required (1) to specify input assumptions and values based on transparent review of empirical evidence and (2) to report (a) increases in utilization and costs due to improved insurance/access, (b) savings due to simplified payment administration (a single payment process using one set of coverage and reimbursement rules), lower drug prices, and other specified reasons, and (c) total system costs and net costs of the single-payer plan.

For this report, we did not require or consider financing (revenue) plans, which turn on an entirely different set of technical issues. We also did not seek analyses of broader economic effects, such as de-investing in the private insurance market or facilitation of labor mobility and start-ups through delinking of insurance and employment. Our analysis also omits long-term effects on medical innovation.

Studies were reviewed by at least 2 team members before finalizing inclusion or exclusion. Uncertain decisions (e.g., regarding adequacy of technical information or severity of deviation from the study definition of single-payer) were discussed with the entire team.

### Extraction

We extracted the following information from each study: annual healthcare costs without single-payer (specified for the year and setting, at the national or state level), initial-year annual cost under single-payer, cost increase due to utilization growth, and savings (from all sources and 4 specific categories: simplified payment administration, lowered costs for medications [and for durable medical equipment, if bundled together], reduced clinical inefficiency [i.e., unneeded procedures] and fraud, and a switch to Medicare payment rates, which are lower than private insurance rates). We did not report transition costs such as purchases of for-profit businesses and training (which were, in any case, rarely assessed), and no study quantified the costs of potential first-year implementation challenges. If available, we extracted longer term costs and savings, defined as costs or savings accumulated subsequent to the first year of implementation. We also extracted or calculated the utilization increase assumed for newly insured individuals.

Each study was reviewed by 2 team members, and all study extractions were reviewed by the senior investigator (JGK), who requested refinements and further documentation for unclear or unexpected values. When we had questions due to omissions or ambiguity in the report, we attempted to contact study authors. We also sent them, when successfully located, a report draft for review.

### Analysis

We standardized all cost numbers to percentage of contemporaneous total health system costs, to allow for direct comparison across times and locations. This approach obviated the need for inflation adjustments. We standardized costs due to increased utilization as the increase in annual cost for the newly insured divided by the mean cost for the already insured. We examined results visually, ordered by year and by net cost (highest net cost to highest net savings).

To assess the association of net cost with plan and analysis features (e.g., whether drug price reductions were considered), we used a visual method (color-coding analysis features). We also conducted univariate and multivariate linear regressions with net savings or cost as the outcome and with the following predictors: utilization increase, specific savings categories, type of funder organization, and type of analyst organization. In the multivariate analysis, we assigned dummy variables for missingness of the utilization predictor.

## Results

### Studies identified

We reviewed 90 studies and included primary analyses of 22 single-payer plans from 18 studies, published between 1991 and 2018, including 8 national and 14 state-level plans (Massachusetts, California, Maryland, Vermont, Minnesota, Pennsylvania, New York, and Oregon). Included studies are listed in Table F in S1 Appendix. Analysis teams included US government agencies, business consultants and research organizations, and academics. Nine single-payer plans (from 6 studies) were excluded for the following reasons: age limits on single-payer, varied benefits across individuals, balance billing, inclusion of private insurers or intermediaries in the plan or analysis, and lack of specification of assumptions regarding utilization and savings. Twelve studies were not reviewed because of duplication (same author, different state, earlier, *n* = 11) and age (1971, *n* = 1).

### Projected costs and savings

**Fig 2:**
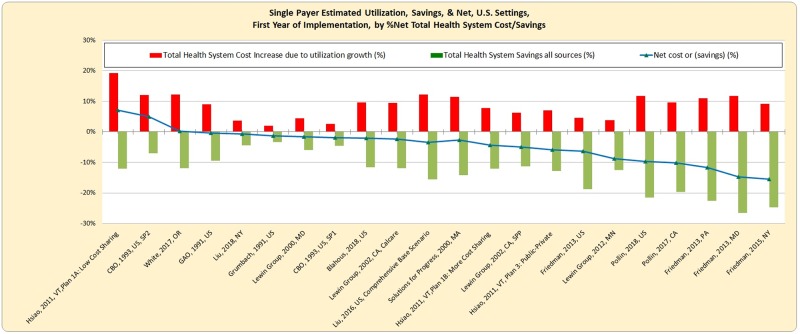
Net savings for single-payer in first year of implementation, sorted by net cost/savings. The median finding was savings (−3.46% of total health system costs), and analyses of 19 of 22 plans found net savings.

Net cost or savings in the first year of single-payer operation varies from an increase of 7.2% of system costs to a reduction of 15.5% (Fig 2). The median finding was a net savings of 3.5% of system costs, and analyses of 19 of 22 plans found net savings. Net costs reflect the balance of added costs due to higher utilization (by eliminating uninsurance and in some studies also capturing the increase due to ending underinsurance) and savings (via payment simplification, lower drug prices, and other factors). Higher utilization increases costs by 2.0% to 19.3% (median 9.3%). Total savings range from 3.3% to 26.5% (median 12.1%).

The cost increase due to expansion of insurance coverage varies due to the number of newly covered individuals and generosity of coverage benefits, but also reflects policy components and expert assessment. For example, study estimates of increased utilization by newly covered individuals range from 25% to 80% of the costs for those already insured, reflecting varied assessments of uninsured individuals’ healthcare access and health status. Additionally, cost-control choices such as copays vary across plans.

**Fig 3:**
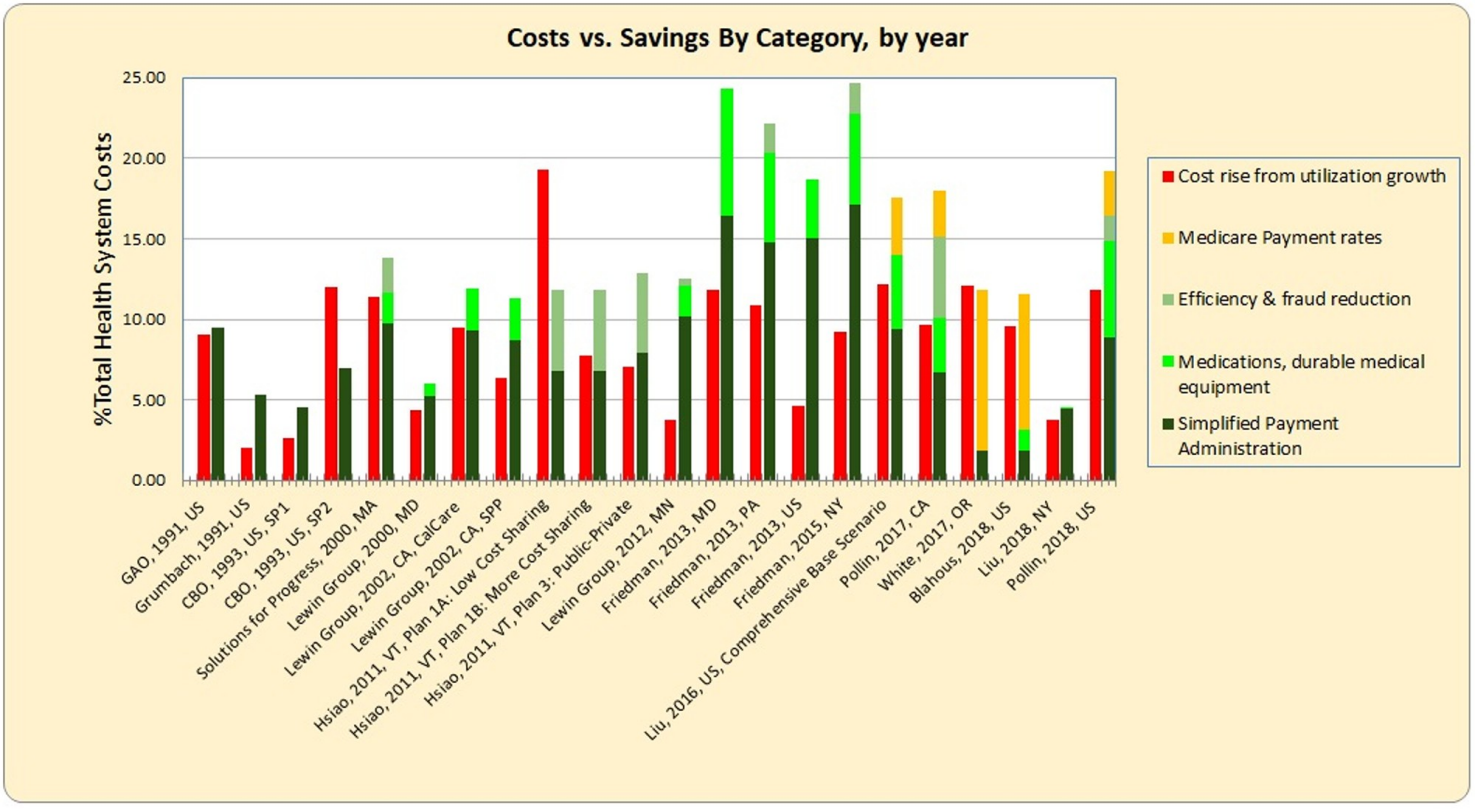
Costs versus savings for single-payer by category. Plans listed in order by year. Simplified payment administration was the greatest source of savings, for a median of 8.8%.

The mix of projected savings from single-payer shows both consistent and variable elements across studies (Fig 3). All studies estimate lower costs due to simplified payment administration, but vary in the size of these savings and in the inclusion and magnitude of other savings. Administrative savings vary from 1.2% to 16.4% (median 8.8%) of healthcare spending. Savings from lowered prices for medications and durable medical equipment are included in 12 models and range from 0.2% to 7.9%. Savings from reduced fraud and waste are included in 10 models and range from 0.4% to 5.0%. Savings due to a shift to Medicare payment rates are included in 8 models and range from 1.4% to 10.0%. Over time, utilization increases are stable and projected savings grow, leading to larger estimates for potential savings.

In the long term, projected net savings increase, due to a more tightly controlled rate of growth. For the 10 studies with projections for up to 11 years, each year resulted in a mean 1.4% shift toward net savings (Text A and Figs A and B in S1 Appendix). At this rate, the 3 studies that find net costs in the first year would achieve net savings by 10 years.

### Influence of plan and analysis features on findings

**Fig 4:**
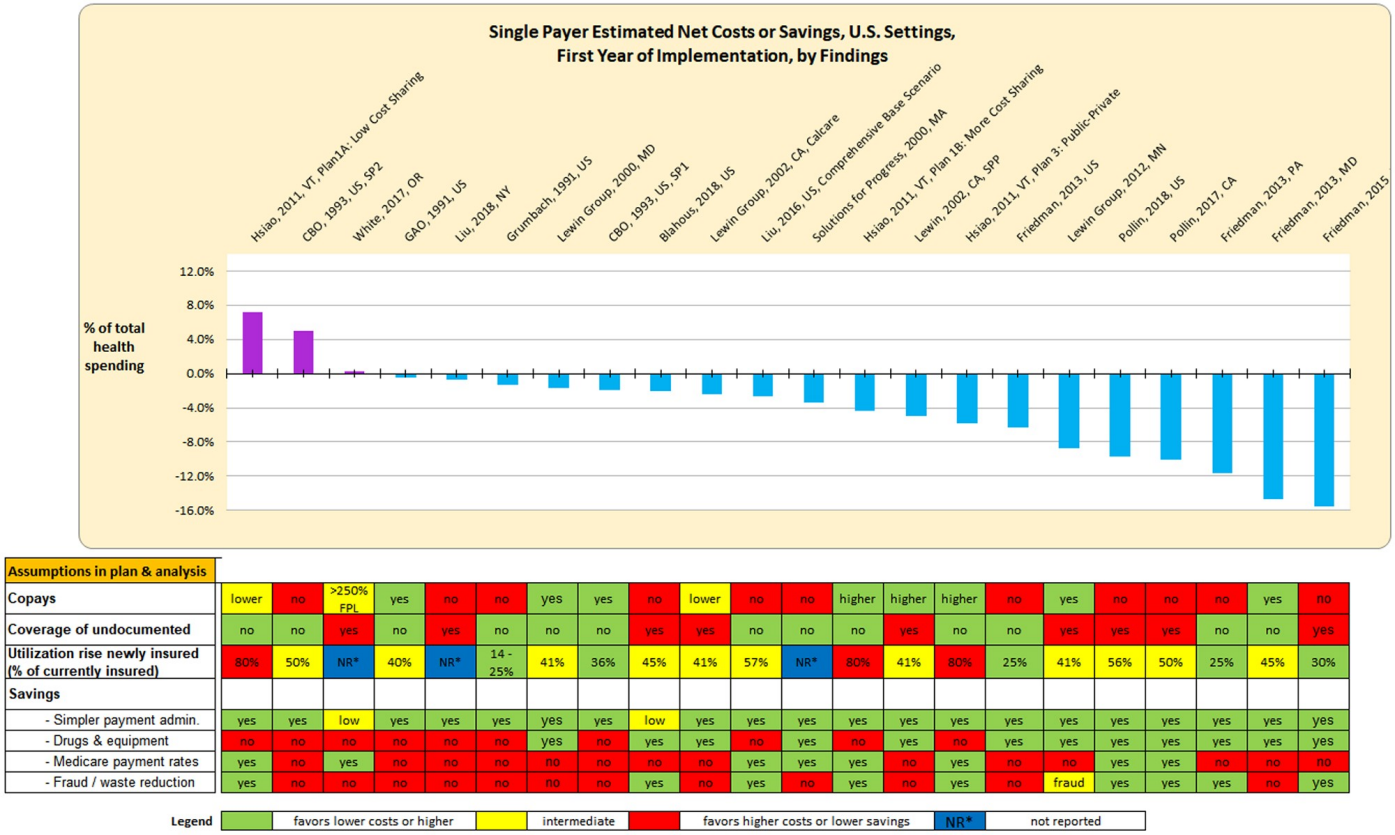
Net costs or savings versus assumptions in plans and analyses, sorted by net costs/savings. The 3 models that found net costs in the first year (Hsiao 2011 Low Cost Sharing, CBO 1993 SP2, and White 2017) shared specific policy choices including low or no cost sharing (copays), rich benefit packages, and a lack of savings captured from reduced medication/medical equipment costs.

Fig 4 presents net costs or savings alongside a color-coded summary of key plan features and model assumptions. The 3 of 22 models that found net costs in the first year shared specific policy choices including low or no cost sharing (copays), rich benefit packages, and a lack of savings predicted from reduced medication/medical equipment costs. Two of these models (Hsiao 2011 Low Cost Sharing and CBO 1993 SP2) are estimated for additional scenarios that yield net savings.

**Fig 5:**
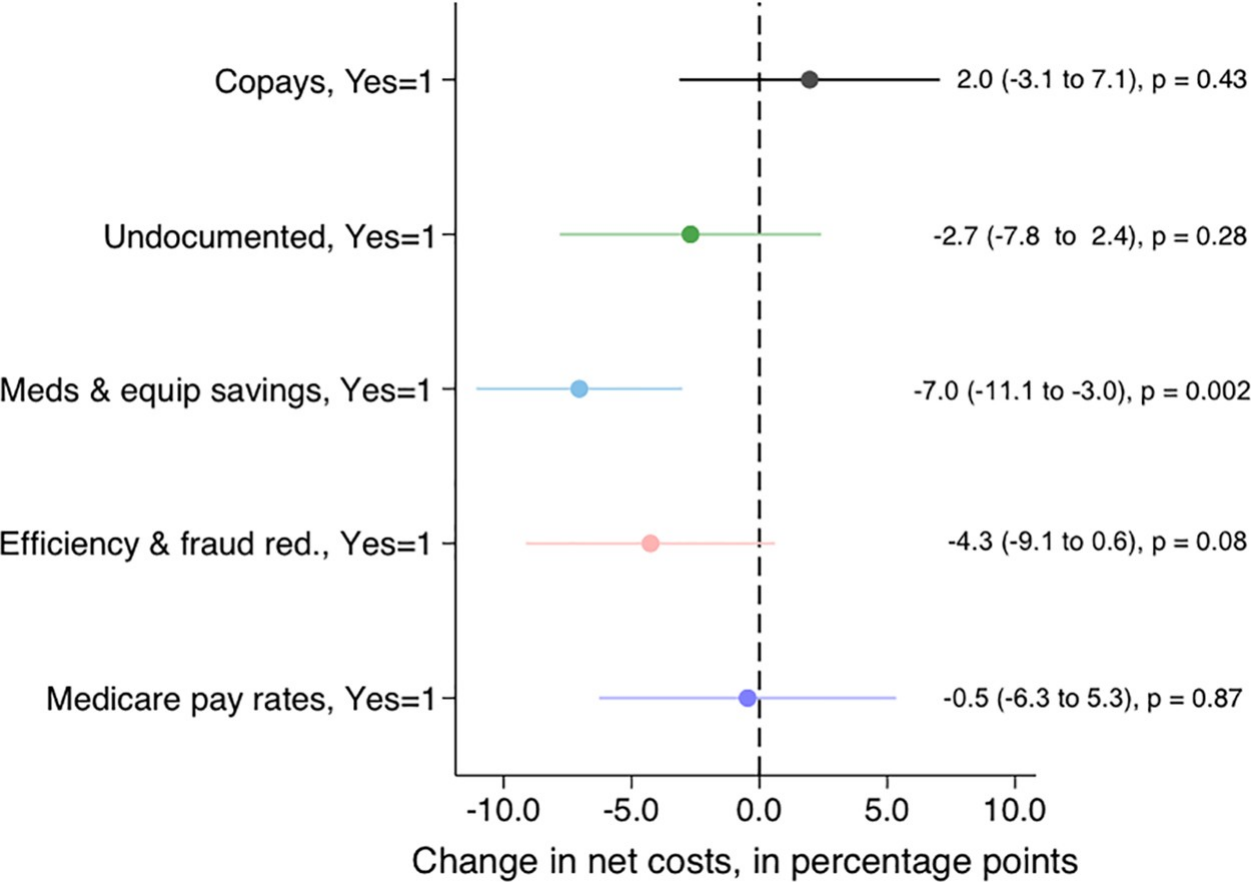
Net costs versus the inclusion of different analysis features. Each estimate comes from a separate linear regression of net costs and a binary predictor. Error bars represent 95% confidence intervals.

We next assessed whether the inclusion of different analysis features (yes or no) was associated with net costs, based on univariate regressions (Fig 5). Cost sharing did not have a significant association with net costs across all studies (2.0 points, 95% CI −3.1 to 7.1, *p* = 0.43); 11 of 19 analyses showing net savings in the first year included no or low cost sharing in their plans. Similarly, the association between inclusion of undocumented individuals and net costs was not statistically significant (−2.7 points, 95% CI −7.8 to 2.4, *p* = 0.28). Inclusion of medication and equipment savings in the model was associated with lower net costs by 7.0 points (95% CI −11.1 to –3.0, *p* = 0.002), and inclusion of efficiency gains and fraud reduction was associated with lower net costs by 4.3 points but not significant (95% CI −9.1 to 0.6, *p* = 0.08). Inclusion of a shift to Medicare payment rates was not a strong predictor of net costs. We cannot assess the association between net costs and presence or absence of administrative savings in these dichotomous analyses because all studies include these savings. The number of different analysis features included in the model was also associated with lower net costs. For each additional analysis feature included, net costs were reduced by 2.3 points (95% CI –4.3 to –0.3, *p* = 0.02).

**Fig 6:**
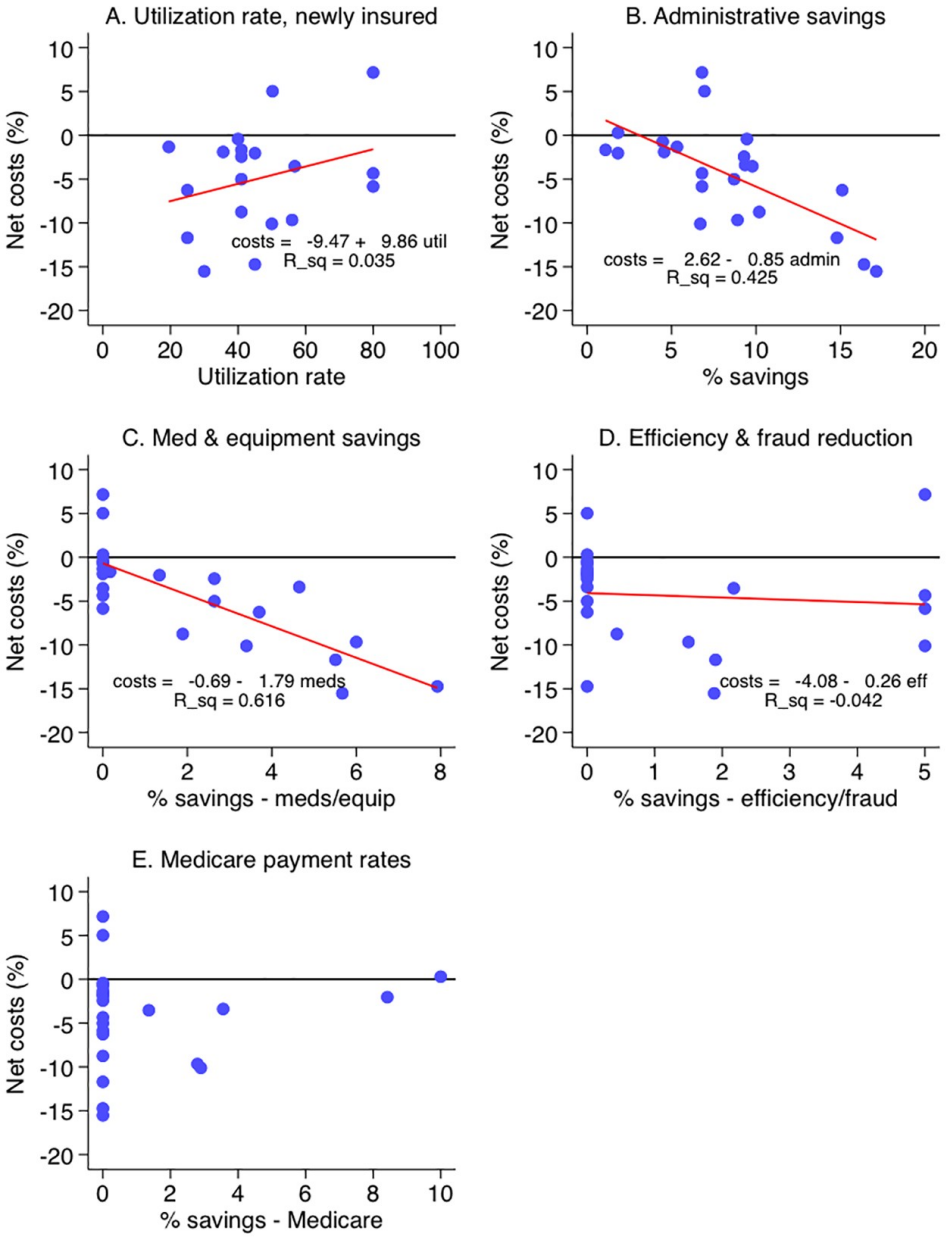
Net cost (%) versus utilization rise and savings magnitude. (A) Utilization rate; (B) administrative savings; (C) medicine and equipment savings; (D) efficiency gains and fraud reduction; (E) Medicare payment rate. Each dot represents 1 model. The red lines represent linear regressions, with displayed results indicating the regression equation (including intercept and slope) and *R*^2^ (proportion of variation explained). The regression line for Medicare payment rate (E) was omitted due to the preponderance of 0 values (73%, or all but 6, of the 22 models). Higher utilization was associated with greater costs, whereas the magnitude of administrative and medication/equipment savings was associated with reduced net costs.

In univariate regressions of net savings against the magnitude of inputs, several relationships emerge (Fig 6). A 1-point increase in utilization rate was associated with higher net costs of 9.9 points; however, this relationship did not reach statistical significance (95% CI −6.3 to 26.0, *p* = 0.22). In contrast, the magnitude of net savings was associated with higher savings in administrative costs (net cost −0.85 points, 95% CI −1.3 to −0.4, *p* = 0.01) and in medication and equipment costs (−1.79 points, 95% CI −2.43 to −1.16, *p* < 0.0001). Net savings were not strongly related to Medicare payment rates or efficiency gains/fraud reduction.

In a multivariate regression (limited by small sample size), we found that net costs were associated with medication and equipment cost savings (−1.5 points, 95% CI −2.6 to −0.4, *p* = 0.01); other analysis features did not strongly predict net costs. Lower net costs were associated with funder type (left-leaning versus right-leaning: −6.7 points, 95% CI −11.5 to −1.8, *p* = 0.009) and analyst type (academic versus other: 7.6 points, 95% CI 0.4 to 14.9, *p* = 0.04) in bivariate regressions, but not in multivariate regressions, perhaps due to reduced precision due to the sample size. Tables H and I in S1 Appendix report the multivariate regression details.

## Discussion

We identified 22 credible economic models of the cost of single-payer financing in the US, from a variety of government, business consultant, and academic organizations. We found that 19 (86%) predict net savings in the first year of operations, with a range from 7% higher net cost to 15% lower net cost. Increases in cost due to improved insurance coverage and thus higher utilization were 2% to 19%. Savings from simplified payment administration at insurers and providers, drug cost reductions, and other mechanisms ranged from 3% to 27%. The largest net savings were for plans with reductions in drug costs. Net savings accumulate over time at an estimated 1.4% per year. Of note, we excluded 2 widely publicized studies [20,24], both of which found net costs, on the grounds that these studies made assumptions that included private insurance intermediaries (i.e., not a single-payer) or lacked technical detail for evaluation.

These analyses suggest that single-payer can save money, even in year 1, incorporating a wide range of assumptions about potential savings. More aggressive measures to realize cost reductions are projected to yield greater net savings. This implies that concerns about health system cost growth with single-payer may be misplaced, though costs to government are likely to grow as tax-based financing replaces private insurance premiums and out-of-pocket spending.

### Empirical evidence for model assumptions

The results of these economic models depend on input assumptions regarding the effect of single-payer provisions. In particular, the magnitude of net savings reflects the quantitative effects of utilization rises due to increased insurance and savings strategies. Reasonable analysts may differ on these assumptions based on plan features, setting, and evidence available at the time of modeling. There is growing empirical evidence for each provision, which we review below.

Utilization increases due to new and improved insurance drive the cost growth effects of single-payer. There is strong evidence over decades that the newly insured roughly double their healthcare utilization [25–27]. Medicaid expansion under the Affordable Care Act appears to demonstrate a mix of utilization effects [28,29]. Moreover, in a single-payer system, the newly insured may be younger and healthier than the already insured, meaning that utilization may not increase to the levels of the already insured. Evidence on utilization increases for the underinsured are mixed [30–32]. Importantly, there is evidence that when uninsured individuals gain insurance, increases in utilization for the newly insured are balanced by slightly lower utilization for the already insured, due to supply-side constraints [33–35]. However, with a decrease in billing-related administrative burden for clinicians, a 10% or greater rise in physician clinical capacity may occur, which would accommodate additional care utilization. Finally, increases in utilization for the uninsured and underinsured are likely to result in increased use of preventive services, which should lead to some future cost saving [25,36].

Simplified payment administration represents the largest type of savings from single-payer. There is very strong evidence that billing and insurance-related administrative burden is higher in the US than in Canada (which has single-payer) by 12%–15% of total healthcare costs [13]. The excess administrative costs are split roughly 50% at insurers and 50% at providers. Studies of hospitals find consistent large differences in administrative costs between the US and single-payer systems in Europe [37]. There is no direct evidence of ability to capture all of this excess, but solid empirical data from Canada and other Organisation for Economic Co-operation and Development (OECD) countries support the intuition that administrative costs would sharply decrease with elimination or streamlining of existing onerous payment processes.

Lower drug spending is typically the second largest source of savings with single-payer, and predicts large net savings. The US Veterans Administration (VA) gets a 30% discount on prescription medications compared to private Medicare Advantage Plans [38,39]. US per-capita drug spending exceeds that of any other country [38,39]. Drug prices are the primary driver of higher cost, with the US spending $1,011 annually per capita on prescription drugs compared to the OECD average of $422 [11].

Research estimates savings of 30% for diabetes drugs through use of drug formularies, due to medication choice and prices [40]. Drug companies argue that reducing prices will reduce research and innovation. However, many more expensive drugs offer limited medical benefits [38,41,42]. Further, drug firms often raise prices after recovering development costs. Research and development costs for 10 companies that launched new cancer agents were $9 billion, while revenue exceeded $67 billion [43]. Perhaps most tellingly, Fortune 500 drug companies had a mean profit reported in 2019 of 24% compared to 9% for all corporations [38,44,45]. Drug companies claim that if the entire health system gets the same discount as the VA, the discount levels will substantially decrease. However, if Medicare adopted the VA’s tighter drug formulary, the savings would be roughly $505 per capita annually [46]. Overall, there is strong evidence of the potential for a substantial reduction in drug costs, with magnitude likely a function of political choices and dynamics. A portion of these savings could also be realized if the government negotiated for lower drug prices in the existing Medicare program.

Reports estimate that up to 20%–40% of US healthcare spending is fraudulent or wasteful [47,48]. However, there is little evidence on how to avoid this spending. The Affordable Care Act set up accountable care organizations (ACOs), groups of healthcare providers responsible for a defined set of patients and contracting with a payer (usually Medicare) for a payment structure tied to performance metrics, in an effort to reduce costs. Recent ACO demonstration projects found minimal savings, potentially less than the cost of administering programs, leading to overall net 0 savings [49]. ACOs that are “two-sided” (using both penalties and shared savings) reduce service costs by a mean of 0.7% yet require on average about 2% costs to administer [50,51]. Overall, between 2013 and 2017, ACOs increased total costs to Medicare by 70 billion when bonuses were taken into account [52]. Recent analysis suggests modestly growing savings, in physician if not hospital groups, potentially more than administration costs [53,54]. Single-payer may facilitate efforts to reduce fraud and waste by providing comprehensive and consistent clinical encounter data within the single billing system (including diagnoses and services, as well as clinical outcomes). Thus, single-payer might bolster the marginally effective efforts in this area. Still, the evidence to support large reductions in waste and fraud is tenuous. Furthermore, a reliance on ACO incentive approaches (which require large patient panels and specific payment structures) could undermine desired features of a single-payer program, such as free choice of provider, substantial use of fee-for-service billing in some plans, and hospital global budgeting. In light of these uncertainties, most economic models do not anticipate reductions in fraud or waste, and those that do generally assume only a modest reduction.

### Limitations

Our analysis has several important limitations. First, the included economic studies varied in methodological rigor and quality of reporting, funding sources, political motivations, and amount of evidence cited to support claims. Although we tried to classify studies by major single-payer and analysis characteristics, uncaptured variations may have added noise in the comparison. Relatedly, the diversity of plans under study did not allow for a formal meta-analysis, which is designed to integrate empirical evaluations of standardized interventions, especially using measures of association such as odds ratios.

Second, we did not apply quality rating scores for the included economic studies. We found no quality rating scores for health system modeling, as existing scores are intended for evaluation studies, empirical measurements of costs and effects, or decision analyses [55–57]. A quality rating system could be useful. Included studies all lacked sensitivity analyses, and the selection of the most appropriate data source for input values could be subjective. For example, studies varied in what percentage of savings could be achieved through simplification of payment administration. We are unaware of studies analyzing the effects of other key inputs, such as reductions in reimbursement rate. Future research is needed to assess the quality of single-payer studies, analyze key model inputs, and analyze proposed ranges for sensitivity analyses. In terms of the potential for financial conflict of interest bias, we were reassured that a prominent health business consultant (Lewin Group, with several included analyses), presumably with clients that stand to lose money with single-payer, nonetheless found net savings.

Third, no single-payer system has been implemented in the US, due to lack of government approval even for demonstration projects. Thus, there is no domestic, large-scale empirical example to properly test the economic models. Much of the research on single-payer is based on evidence from single-payer nations such as Canada, Australia, and Taiwan. As reviewed above, US health systems that approximate single-payer, such as the VA, and other empirical studies provide support for model assumptions. Ultimately, our goal was not to compare cost models with (nonexistent) empirical benchmarks, but to assess the consistency of inputs across models and with empirical evidence, and to characterize patterns in model findings. Assuming that US single-payer demonstrations are coming, economic models can be tested and refined. Until then, the relative consistency of existing models is the best evidence available.

Fourth, our study was limited to proposals of single-payer as defined in the US, with a single (government) payer, and meeting specified criteria. Our results are not generalizable to multi-payer “universal coverage” reforms, which would likely show substantially smaller savings and thus increases in net cost [58]. The Maryland all-payer model, for example, showed 2.7% savings after 3 years, a figure that is significantly lower than the average savings from single-payer systems we found in our review [59]. Multi-payer systems have higher costs in part due to increased cost shifting. Our analysis is not able to quantify precisely the effects of reduced cost sharing. A unified provider payment system, as opposed to a single-payer system, may accomplish substantial cost savings, but our analysis only considered the latter. Indeed, many OECD countries have a unified payment system with a standard benefits package, a single payment process, a single formulary, and not-for-profit insurers, which shares many features with “single-payer.” Finally, despite the drawbacks of our narrow inclusion criteria, a benefit is that our results provide a clearer and more relevant assessment of the economic impact of a single-payer system in the US.

Fifth, in addition to saving costs, unified payment models such as single-payer have the potential to foster quality and efficient care through payment signals, as well as to monitor trends in care patterns via rapid access to highly standardized claims data. For example, in Japan’s unified payment system, price incentives are used to promote public health goals, such as increasing preventive care [60]. The use of price incentives to drive performance is common in high-income countries [61]. However, studies did not include this in their analysis, so we deemed it outside the scope of our study.

Sixth, as with any review, our search period is time-limited, ending in December 2018. We are aware of 1 study in 2019 [62], but did not systematically search for other studies. We limited our Google searches to 10 pages. However, we never found a relevant study after page 2 of search results, increasing our confidence that a 10-page review was adequate. We will update this analysis in coming years.

Finally, we examined only economic studies of system operating costs, in the first year and over time. We ignored one-time transition costs (in particular, purchase of for-profit entities, unemployment and pension benefits, and retraining of displaced workers). Informal review of existing evidence suggests that these costs are small in comparison to health system spending, which is 18% of the economy. We also did not examine financing, e.g., taxation strategies. These are important next steps.

### Policy implications

This review highlights a high degree of analytic consensus that single-payer financing would result in a favorable outcome for system financial burden: efficiency savings exceed added costs. A net cost reduction of 3%–4% is likely initially, growing over time. Net savings would be expected to occur, if not immediately, certainly within a few years. However, maximizing performance and savings will require optimized implementation. Payment procedures must be as simple as in other countries, drug prices a substantial reduction from contemporary levels, and comprehensive clinical data used in sophisticated ways to identify and reduce inappropriate care. The logical next step is real-world experimentation, including evaluation and refinement to minimize transition costs and achieve modeled performance in reality.

## Supporting information

S1 Appendix Additional detail on methods and findings. (DOCX)

## Abbreviations

ACO: accountable care organization
OECD: Organisation for Economic Co-operation and Development
VA: US Veterans Administration
