# Supplementary material for: Projected costs of single-payer healthcare financing in the United States: A systematic review of economic analyses
Source: PLoS Med. 2020 Jan 15;17(1):e1003013. doi: 10.1371/journal.pmed.1003013 (PMC6961869; doi:10.1371/journal.pmed.1003013)
Supplement: S1 Appendix — (DOCX) [file pmed.1003013.s001.docx]

**S1. Supporting Information**

Projected Costs of Single-Payer Health Care Financing in the United States:
a Systematic Review of Economic Analyses

| **Table A. Definitions** |
| --- |
| **Net Savings** = **Savings** (from Simplified Payment Administration, Drug Price Reductions, Lower (Medicare) Reimbursement Rates, Fraud & Waste Reduction) *minus* **Added Cost** (from Higher Utilization due to Universal Comprehensive Insurance)  **Net Costs**= A term we use if Net Savings are negative.  **Simplified Payment Administration**= Savings due to a single payment portal using a single set of reimbursement (or billing) rules and procedures.  **Drug Cost Reductions** = Savings due to reductions in drug price, most commonly achieved through negotiations with suppliers and a formulary. Also referred to as medications, and in some analyses including equipment.  **Fraud and Waste Reduction**= Savings due to reduction in fraudulent or wasteful practices, such as “upcoding”, duplicative services, defensive medicine, etc. |

| **Table B. Search Strategy** | |
| --- | --- |
| Source | Method |
| **Known compilations of single-payer studies** | All studies listed on the pro-single-payer website [HealthcareNow](https://www.healthcare-now.org/single-payer-studies/listing-of-single-payer-studies/) were screened for inclusion  All Studies on the Physicians for a National Health Program [website](http://pnhp.org/how-much-would-single-payer-cost/) were screened for inclusion  For completeness, we searched Partnership for America’s Health Care Future (coalition of insurers and others), and found no single-payer studies. |
| **Convenience Sample of experts** | We inquired with a convenience sample of single-payer, policy, and health economics experts: David Himmelstein, Stephanie Woolhandler, Adam Gaffney, Kevin Grumbach, William Hsiao, Justin White, Colin Boyle , Robert Pollin. |
| **Pubmed search** | Economic analyses of single-payer studies are not routinely published in the peer reviewed literature. We conducted a Pubmed search which did not return any relevant studies.  The following terms were placed into a pubmed advanced search in October 2018   1. Single-payer[ti] OR Single-payer systems [MeSH] 2. cost*[ti] OR model*[ti]  OR economic *[ti]   40 results were reviewed for inclusion, first by title. If the title was relevant, abstract was then reviewed. If the abstract was relevant, full text and citations were reviewed. One citation was not available online (NYS DoH, below). No papers passed inclusion criteria.  New York State Department of Health, Division of Planning, Policy and Resource Development. A proposal for Universal New York Health Care (UNY*Care). September 1, 1989.  The search terms were then expanded, but returned 4183 studies on 12/28/18 and reviewing all studies was deemed beyond the scope of this paper.   1. single-payer system  [MeSH] OR Single-payer systems [MeSH] OR National Health Program[MeSH] OR single-payer [tw] OR single-payer [tw] 2. single-payer system  [tiab] OR Single-payer systems [MeSH] OR National Health Program[MeSH] 3. cost*[ti] OR model*[ti]  OR economic *[ti] 4. #1 AND #2 |
| Google Scholar Search | The following terms were entered into Google Scholar in order to search for studies that had been published in the “gray literature”  Intext: (“Single-payer” OR “single-payersingle payer”) AND (“cost” OR “model” OR “economic” OR  “cost-benefit”)  The first 10 pages of search results were reviewed on September 4th 2019. Search results were screened by title and abstract. Studies were either immediately available via search, mentioned in the full text of a search result, or cited in the search result. Two studies were reviewed for inclusion, both of which had already been included (Pollin 2017, Mercatus 2018). Google Scholar and Google Search are imperfect tools for completing a systematic literature review as the search algorithm is frequently updated, and different results are produced based on a user’s prior search history and location. We take these limitations seriously, which is why we used other databases as our primary search tools. Nonetheless, we chose to use Google Scholar and Google Search as supplemental sources as they are important sources of grey literature / non-peer-reviewed publications such as the economic analyses in this study, that can add to the comprehensiveness of the study (Haddaway NR, Collins AM, Coughlin D, Kirk S. The role of Google Scholar in evidence reviews and its applicability to grey literature searching. PLoS ONE. 2015;10(9):e0138237. doi: 10.1371/journal.pone.0138237.) It was not feasible to review all of the Google Scholar results (>24,000) and Google Search results (>1 million), therefore we limited our review to the first 10 pages given the low yield of analyses found in this range. We also sought to diminish the bias associated with a personalized search by logging out from Google accounts. |
| Google Search | The following terms were entered into Google in order to search for studies that had been published in the “gray literature”  Intext: (“Single-payer” OR “single-payersingle payer”) AND (“cost” OR “model” OR “economic” OR  “cost-benefit”)  The first 10 pages of search results were reviewed on October 16th 2018. Search results were screened by title and abstract. Studies were either immediately available via search, mentioned in the full text of a search result, or cited in the search result. Four studies were reviewed for inclusion. Of those, 2 had already been included (Grumbach et al 1991, CBO 1991), 1 was added (Lewin 2001 Maryland), and one full text could not be identified (Meyer, JA, Silow- Carrol , S and Sullivan S. A National Health Plan in the US: The Long-term Impact on Business and the Economy (Reston VA: Economic and Social Research Institute)  See above for a discussion of the limitations of using Google Search in a systematic review. |
| *Table B. Search Strategy. We selected studies for review through several steps. First, we searched existing lists of single-payer economic studies online. Second, we surveyed a convenience sample of policy experts to identify studies missing from these lists. Finally, we conducted Google and Pubmed searches.* | |

| **Table C. Studies Excluded Based on Criteria** | | | |
| --- | --- | --- | --- |
| **Study** | **Plans** | **Reason(s) Excluded** | |
| [The Price of single-payer: A Fiscal and Economic Analysis of the New York Health Act](https://www.healthcare-now.org/single-payer-studies/new-york-freopp-2017-2/) | 1 | Critique of Friedman analysis and no new economic modeling | |
| [ColoradoCare: An Independent Analysis](https://www.healthcare-now.org/single-payer-studies/colorado-health-institute-2016/) | 1 | Assessed financing but not cost | |
| [Economic Analysis of the ColoradoCare Proposal](https://www.healthcare-now.org/single-payer-studies/colorado-foundation-for-universal-health-care-2015/) | 1 | Assessed financing but not cost | |
| [Green Mountain Care: A Comprehensive Model for Building Vermont’s Universal Health Care System](https://www.healthcare-now.org/single-payer-studies/vermont-agency-of-administration-2014/) | 1 | Assessed financing but not cost | |
| [Achieving Quality, Affordable Health Insurance for All New Yorkers: An Analysis of Reform Options](https://www.healthcare-now.org/single-payer-studies/new-york-urban-institute-2009/) | 4 | Not a true single-payer (would retain Medicaid and CHIP) | |
| Fiscal analysis of SB 840 | 1 | Insufficient technical detail to understand methods | |
| Quantitative and Comparative Analysis of Reform Options for Extending Health Care Coverage in New Mexico | 5 | Insufficient technical detail to understand methods | |
| [Kansas – Pricing the Roadmap To Health Insurance Reform Options](https://www.healthcare-now.org/single-payer-studies/kansas-schramm-raleigh-2007/) | 5 | Insufficient technical detail to understand methods | |
| [Feasibility of a Single-Payer Health Care Model for the State of Maine](https://www.healthcare-now.org/single-payer-studies/maine-mathematica-2002/) | 1 | Insufficient technical detail to understand methods | |
| [Fiscal estimates of SB 562 The Healthy California Act](https://www.healthcare-now.org/single-payer-studies/california-senate-committee-on-appropriations-2017/) (Committee Staff Report) | 1 | Insufficient technical info and doesn't reflect SP bill | |
| [State of Vermont Health Care Financing Plan Beginning Calendar Year 2017 Analysis](https://www.healthcare-now.org/single-payer-studies/vermont-umass-wakely-consulting-2013/) | 1 | Not a SP system (includes multiple payers - Medicare, Tricare, VA etc.) | |
| [An Analysis of Senator Sanders single-payer Plan](https://www.healthcare-now.org/single-payer-studies/kenneth-thorpe-2016/), Kenneth Thorpe 2016 | 1 | Insufficient technical detail to understand methods | |
| [Impacts of Health Care Reform: Projections of Costs and Savings](https://www.healthcare-now.org/single-payer-studies/kenneth-thorpe-2005/) | 1 | Insufficient technical detail to understand methods | |
| [Universal Coverage: How Do We Pay For It?](https://www.healthcare-now.org/single-payer-studies/economic-policy-institute-1998/) | 1 | Insufficient technical detail. A financing study, not a cost study | |
| [(HR1300 Russo) Estimates of Health Care Proposals from the 102nd Congress](https://www.healthcare-now.org/single-payer-studies/congressional-budget-office-1993-russo/) | 1 | Same analysis of CBO 1993 but for a different year (analyzes 1995 but uses same assumptions) | |
| [Canadian Health Insurance: Estimating Costs and Savings for the United States](https://www.healthcare-now.org/single-payer-studies/government-accounting-office-1992/) | 1 | Repeat of Gao 1991 | |
| [Analysis of Health Insurance Proposals Introduced in the 92d Congress](https://www.healthcare-now.org/single-payer-studies/department-of-health-education-and-welfare/) | 1 | Considered too outdated | |
| Thorpe 2000 Missouri | 2 | Does not meet inclusion criteria: Coverage only <65 | |
| Mathematica Maine 2002 | 3 | Explicit mention of varied levels of benefits, insufficient technical material | |
| Urban Institute 2016 Sanders Plan | 1 | Assumes private intermediaries | |
| Jodi Liu RAND Dissertation 2016 (Catastrophic single-payer Plan) | 1 (1 plan included) | Providers who participate in SP legally accept other payments for covered services | |
| [The Feasibility of Consolidated Health Care Financing and Streamlined Health Care Delivery in Massachusetts](https://www.healthcare-now.org/single-payer-studies/massachusetts-lecg-2002/) | 3 | Assumes role of private insurers | |
| [Action Costs Less: The Health Care Amendment Standards and Options for Reform](https://docs.google.com/viewerng/viewer?url=https://www.healthcare-now.org/wp-content/uploads/2017/08/Massachusetts-Thorpe-2005.pdf&hl=en) | 1 | | Insufficient technical detail to understand methods |
| Total | 24 studies, 40 plans | | |
| *Table C. Excluded Based on Criteria. These studies were excluded based on criteria outlined in our methods section* | | | |

| **Table D. Studies Excluded Based on Redundancy/Age** | | |
| --- | --- | --- |
| **Study** | **Total Plans** | **Plans Meeting Criteria** |
| [Three Possibilities for Colorado’s Future Health Care Financing and Delivery](https://www.healthcare-now.org/single-payer-studies/colorado-friedman-2013-2/) | 3 | 1 |
| [Cost and funding of proposed Medicare for All in Massachusetts bill](https://www.healthcare-now.org/single-payer-studies/massachusetts-friedman-2010/) | 1 | 1 |
| [Cost and Coverage Impacts of Five Proposals to Reform the Colorado Health Care System](https://www.healthcare-now.org/single-payer-studies/colorado-lewin-group-2007/) | 5 | 1 |
| [The Healthcare for All Californians Act: Cost and Economic Impacts Analysis](https://www.healthcare-now.org/single-payer-studies/california-lewin-group-2005/) | 1 | 1 |
| [The Georgia SecureCare Program: Estimated Cost and Coverage Impacts](https://docs.google.com/viewerng/viewer?url=https://www.healthcare-now.org/wp-content/uploads/2017/08/Georgia-Lewin-2003.pdf&hl=en) | 1 | 1 |
| [Rhode Island Can Afford HealthCare for All](https://www.healthcare-now.org/single-payer-studies/rhode-island-solutions-for-progress-2002/) | 3 | 2 |
| [Analysis of the Costs and Impact of Universal Health Care Coverage Under a single-payer Model for the State of Vermont](https://www.healthcare-now.org/single-payer-studies/vermont-lewin-group-2001/) | 1 | 1 |
| [Massachusetts Comparative Projected Health Expenditure Model](https://www.healthcare-now.org/single-payer-studies/massachusetts-lewin-group-1998/) | 1 | 1 |
| [The Financial Impact of Alternative Health Reform Plans in New Mexico](https://www.healthcare-now.org/single-payer-studies/new-mexico-lewin-group-1994/) | 2 | 1 |
| [Preliminary Estimate of the Effects of S. 491, American Health Security Act of 1993 (different efficacy for spending caps need to be extracted)](https://www.healthcare-now.org/single-payer-studies/congressional-budget-office-1993-wellstone/) | 1 | 1 |
| [Universal Health Insurance Coverage Using Medicare's Payment Rates (include three alternative scenarios for 1989)](https://www.healthcare-now.org/single-payer-studies/congressional-budget-office-1991/) | 2 | 1 |
| Total | 21 | 12 |
| *Table D. Studies Excluded Based on Redundancy/Age. These studies had analyses that met inclusion criteria but were excluded based on redundancy with included studies (same analysis teams and comparable SP plans) and relative age (older than the comparable included studies; 2007 or earlier except for two Friedman studies 2010 & 2013). See Table E below for comparison of overall findings for excluded vs. included studies.* | | |

| **Table E. Net Results of Studies Excluded for Redundancy** | | | | |
| --- | --- | --- | --- | --- |
| Studies that meet criteria but omitted as redundant | | | Included studies same analysts | |
|  | # plans | net savings/ costs % | net savings / costs % | # plans |
| Lewin | 4 | -4.48 | -3.32 | 4 |
| Friedman | 2 | -12.05 | -12.63 | 4 |
| CBO | 4 | 1.55 | -5.31 | 2 |
| Solutions for Progress | 2 | -2.70 | -7.50 | 1 |
| *Table E. Net Results of Studies Excluded for Redundancy.* | | | | |

| **Table F. Included Studies (alphabetical)** |
| --- |
| 1. Blahous C (2018). The Costs of a National Single-Payer Healthcare System. Arlington (VA): Mercatus Center, George Mason University; 2018 Jul [cited 2019 Dec 18]. Available from: <https://www.mercatus.org/system/files/blahous-costs-medicare-mercatus-working-paper-v1_1.pdf>. |
| 1. Congressional Budget Office (1993). Single-Payer and All-Payer Health Insurance Systems Using Medicare Payment Rates. Available at <https://www.cbo.gov/publication/16595> (accessed 12/24/2019) |
| 1. Friedman, Gerald (2013). Financing the Maryland Health Security Act. Available at <https://www.healthcare-now.org/wp-content/uploads/2017/08/Maryland-Friedman-2013.pdf> (accessed 12/24/2019) |
| 1. Friedman, Gerald (2013). Funding HR 676: The Expanded and Improved Medicare for All Act: How we can afford a national single-payer health plan. Available at <https://www.healthcare-now.org/single-payer-studies/gerald-friedman-2013/> (accessed 12/24/2019) |
| 1. Friedman, Gerald (2013). The Pennsylvania Health Care Plan Impact and Implementation. Available at <https://www.healthcare-now.org/wp-content/uploads/2017/08/Pennsylvania-Friedman-2013.pdf> (accessed 12/24/2019) |
| 1. Friedman, Gerald (2015). Economic Analysis of the New York Health Act. Available at <http://www.infoshare.org/main/Economic_Analysis_New_York_Health_Act_-_GFriedman_-_April_2015.pdf> (accessed 12/24/2019) |
| 1. Government Accounting Office (1991). Canadian Health Insurance: Lessons for the United States. Available at <https://www.gao.gov/products/T-HRD-91-35> (Accessed 12/24/2019) |
| 1. Grumbach, K., Bodenheimer, T., Himmelstein, D. U., & Woolhandler, S. (1991). Liberal benefits, conservative spending: the Physicians for a National Health Program proposal. *JAMA*, *265*(19), 2549-2554. Available at <https://jamanetwork.com/journals/jama/article-abstract/385948> (Accessed 12/24/2019) |
| 1. Hsiao, W., Kappel, S., & Gruber, J. (2011). Act 128: Health system reform design. achieving affordable universal health care in Vermont. *Final Report to the Vermont Legislature (February 19, 2011)*. Available at <https://hcr.vermont.gov/sites/hcr/files/FINAL_REPORT_Hsiao_Final_Report_17_February%202011_3.pdf> (Accessed 12/24/19) |
| 1. Lewin Group (2000). Analysis of the Costs and Impact of Universal Health Care Models for the State of Maryland: The Single-Payer and Multi-Payer Models. Available at <https://www.healthcare-now.org/wp-content/uploads/2017/08/Maryland-Lewin-Group-2000.pdf> (Accessed 12/24/2019) |
| 1. Lewin Group (2002). Cost and Coverage Analysis of Nine Proposals to Expand Health Insurance Coverage in California. *Prepared for The California Health and Human Services Agency*. March 31, 2002. Available at <https://www.healthcare-now.org/wp-content/uploads/2017/08/California-Lewin-2002.pdf> (accessed 12/24/2019) |
| 1. Lewin Group (2012). Cost and economic Impact Analysis of a Single-Payer Plan in Minnesota. Available at <https://www.healthcare-now.org/single-payer-studies/minnesota-lewin-group-2012/> (Accessed 12/24/2019) |
| 1. Liu, Jodi L., Exploring Single-Payer Alternatives for Health Care Reform. Santa Monica, CA: RAND Corporation, 2016. <https://www.rand.org/pubs/rgs_dissertations/RGSD375.html>. |
| 1. Liu, Jodi L., Chapin White, Sarah A. Nowak, Asa Wilks, Jamie Ryan, and Christine Eibner, An Assessment of the New York Health Act: A Single-Payer Option for New York State. Santa Monica, CA: RAND Corporation, 2018. <https://www.rand.org/pubs/research_reports/RR2424.html>. |
| 1. Pollin, R., Heintz, J., Arno, P., & Wicks-Lim, J. (2017). *Economic analysis of the Healthy California single-payer health care proposal (SB-562)*. Political Economy Research Institute, University of Massachusetts. Available at <https://www.peri.umass.edu/publication/item/996-economic-analysis-of-the-healthy-california-single-payer-health-care-proposal-sb-562> (Accessed 12/24/2019) |
| 1. Pollin, R., Heintz, J., Arno, P., Wicks-Lim, J., & Ash, M. (2018). Economic Analysis of Medicare for All. *Political Economy Research Institute, University of Massachusetts: Amherst, MA, USA*. Available at <https://www.peri.umass.edu/publication/item/1127-economic-analysis-of-medicare-for-all> (Accessed 12/24/20149) |
| 1. Solutions for Progress (2000). Massachusetts Can Afford Health Care for All. Available at <https://www.bu.edu/sph/files/2012/07/UHC-1-Nov-00-FINAL.pdf> |
| 1. White, Chapin, Christine Eibner, Jodi L. Liu, Carter C. Price, Nora Leibowitz, Gretchen Morley, Jeanene Smith, Tina Edlund, and Jack Meyer, A Comprehensive Assessment of Four Options for Financing Health Care Delivery in Oregon. Santa Monica, CA: RAND Corporation, 2017. <https://www.rand.org/pubs/research_reports/RR1662.html>. |

| **Table G. PRISMA 2009 Checklist** | | | | |
| --- | --- | --- | --- | --- |
| Section/topic | # | Checklist item | Reported in section | Quote |
| TITLE | | |  |  |
| Title | 1 | Identify the report as a systematic review, meta-analysis, or both. | Title | Projected Costs of Single-Payer Health Care Financing in the United States:  a Systematic Review of Economic Analyses |
| ABSTRACT | | |  |  |
| Structured summary | 2 | Provide a structured summary including, as applicable: background; objectives; data sources; study eligibility criteria, participants, and interventions; study appraisal and synthesis methods; results; limitations; conclusions and implications of key findings; systematic review registration number. | Abstract | Entire abstract |
| INTRODUCTION | | |  |  |
| Rationale | 3 | Describe the rationale for the review in the context of what is already known. | Introduction | Paragraphs starting “Economic analyses are crucial for formally estimating the net cost of single-payer proposals…” and “However, these analyses are complex and heterogeneous, making generalizations difficult….” |
| Objectives | 4 | Provide an explicit statement of questions being addressed with reference to participants, interventions, comparisons, outcomes, and study design (PICOS). | Introduction | The goal of this study is to systematically review economic analyses of the cost of single-payer proposals in the United States (both national and state-level), summarize results in a logical but accessible manner, examine the association of findings with plan features and with analytic methods, and finally to examine the empirical evidence regarding key study assumptions. |
| METHODS | | |  |  |
| Protocol and registration | 5 | Indicate if a review protocol exists, if and where it can be accessed (e.g., Web address), and, if available, provide registration information including registration number. | n/a | n/a |
| Eligibility criteria | 6 | Specify study characteristics (e.g., PICOS, length of follow-up) and report characteristics (e.g., years considered, language, publication status) used as criteria for eligibility, giving rationale. | Methods | Section “Inclusion & Exclusion” |
| Information sources | 7 | Describe all information sources (e.g., databases with dates of coverage, contact with study authors to identify additional studies) in the search and date last searched. | Supporting Information | S5 Table |
| Search | 8 | Present full electronic search strategy for at least one database, including any limits used, such that it could be repeated. | Supporting Information | S5 Table |
| Study selection | 9 | State the process for selecting studies (i.e., screening, eligibility, included in systematic review, and, if applicable, included in the meta-analysis). | Supporting Information | S3 PRISMA Checklist and Flowchart |
| Data collection process | 10 | Describe method of data extraction from reports (e.g., piloted forms, independently, in duplicate) and any processes for obtaining and confirming data from investigators. | Methods | Section “Extraction” |
| Data items | 11 | List and define all variables for which data were sought (e.g., PICOS, funding sources) and any assumptions and simplifications made. | Methods | Section “Extraction” |
| Risk of bias in individual studies | 12 | Describe methods used for assessing risk of bias of individual studies (including specification of whether this was done at the study or outcome level), and how this information is to be used in any data synthesis. | Limitations | Second, we did not apply quality rating scores for the included economic studies. We found no quality rating scores for health system modeling, as existing scores are intended for evaluation studies, empirical measurements of costs and effects, or decision analyses [51, 52, 53]. A quality rating system could be useful. Included studies all lacked sensitivity analyses, and the selection of the most appropriate data source for input values could be subjective. For example, studies varied in what percentage of savings could be achieved through simplification of payment administration. We are unaware of studies analyzing the effects of other key inputs, such as reductions in reimbursement rate. Future research is needed to assess the quality of single-payer studies, analyze key model inputs, and analyze proposed ranges for sensitivity analyses. In terms of potential for financial conflict of interest bias, we were reassured that a prominent health business consultant (Lewin), presumably with clients that stand to lose money with single-payer, nonetheless found net savings. |
| Summary measures | 13 | State the principal summary measures (e.g., risk ratio, difference in means). | Inclusion and Exclusion | For the analysis, all studies were required (1) to specify input assumptions and values based on transparent review of empirical evidence, and (2) to report: (a) increases in utilization and costs due to improved insurance / access, (b) savings due to simplified payment administration (a single payment process using one set of coverage and reimbursement rules), lower drug prices, and other specified reasons, and (c) total system costs and the net costs of single-payer. |
| Synthesis of results | 14 | Describe the methods of handling data and combining results of studies, if done, including measures of consistency (e.g., I^2^) for each meta-analysis. | Analysis | We standardized all cost numbers to percentage of total health system costs, to allow for direct comparison across time and location. This approach obviated the need for inflation adjustments. We standardized utilization costs as the increase in annual cost for the newly insured, divided by the mean cost for the already insured. We examined results visually, ordered by year and by net cost (highest net cost to highest net savings). |

3

| Risk of bias across studies | 15 | Specify any assessment of risk of bias that may affect the cumulative evidence (e.g., publication bias, selective reporting within studies). | Limitations | Second, we did not apply quality rating scores for the included economic studies. We found no quality rating scores for health system modeling, as existing scores are intended for evaluation studies, empirical measurements of costs and effects, or decision analyses [51, 52, 53]. A quality rating system could be useful. Included studies all lacked sensitivity analyses, and the selection of the most appropriate data source for input values could be subjective. For example, studies varied in what percentage of savings could be achieved through simplification of payment administration. We are unaware of studies analyzing the effects of other key inputs, such as reductions in reimbursement rate. Future research is needed to assess the quality of single-payer studies, analyze key model inputs, and analyze proposed ranges for sensitivity analyses. In terms of potential for financial conflict of interest bias, we were reassured that a prominent health business consultant (Lewin), presumably with clients that stand to lose money with single-payer, nonetheless found net savings. |
| --- | --- | --- | --- | --- |
| Additional analyses | 16 | Describe methods of additional analyses (e.g., sensitivity or subgroup analyses, meta-regression), if done, indicating which were pre-specified. | Results | Figs 4-6 |
| RESULTS | | |  |  |
| Study selection | 17 | Give numbers of studies screened, assessed for eligibility, and included in the review, with reasons for exclusions at each stage, ideally with a flow diagram. | Methods | Fig 1 |
| Study characteristics | 18 | For each study, present characteristics for which data were extracted (e.g., study size, PICOS, follow-up period) and provide the citations. | Results | Fig 3 |
| Risk of bias within studies | 19 | Present data on risk of bias of each study and, if available, any outcome level assessment (see item 12). | Limitations | Second, we did not apply quality rating scores for the included economic studies. We found no quality rating scores for health system modeling, as existing scores are intended for evaluation studies, empirical measurements of costs and effects, or decision analyses [51, 52, 53]. A quality rating system could be useful. Included studies all lacked sensitivity analyses, and the selection of the most appropriate data source for input values could be subjective. For example, studies varied in what percentage of savings could be achieved through simplification of payment administration. We are unaware of studies analyzing the effects of other key inputs, such as reductions in reimbursement rate. Future research is needed to assess the quality of single-payer studies, analyze key model inputs, and analyze proposed ranges for sensitivity analyses. In terms of potential for financial conflict of interest bias, we were reassured that a prominent health business consultant (Lewin), presumably with clients that stand to lose money with single-payer, nonetheless found net savings. |
| Results of individual studies | 20 | For all outcomes considered (benefits or harms), present, for each study: (a) simple summary data for each intervention group (b) effect estimates and confidence intervals, ideally with a forest plot. | Results | Fig 1 , 2 |
| Synthesis of results | 21 | Present results of each meta-analysis done, including confidence intervals and measures of consistency. | n/a | n/a |
| Risk of bias across studies | 22 | Present results of any assessment of risk of bias across studies (see Item 15). | Limitations | Second, we did not apply quality rating scores for the included economic studies. We found no quality rating scores for health system modeling, as existing scores are intended for evaluation studies, empirical measurements of costs and effects, or decision analyses [51, 52, 53]. A quality rating system could be useful. Included studies all lacked sensitivity analyses, and the selection of the most appropriate data source for input values could be subjective. For example, studies varied in what percentage of savings could be achieved through simplification of payment administration. We are unaware of studies analyzing the effects of other key inputs, such as reductions in reimbursement rate. Future research is needed to assess the quality of single-payer studies, analyze key model inputs, and analyze proposed ranges for sensitivity analyses. In terms of potential for financial conflict of interest bias, we were reassured that a prominent health business consultant (Lewin), presumably with clients that stand to lose money with single-payer, nonetheless found net savings. |
| Additional analysis | 23 | Give results of additional analyses, if done (e.g., sensitivity or subgroup analyses, meta-regression [see Item 16]). | Results | Figs 4-6 |
| DISCUSSION | | |  |  |
| Summary of evidence | 24 | Summarize the main findings including the strength of evidence for each main outcome; consider their relevance to key groups (e.g., healthcare providers, users, and policy makers). | Discussion | We identified 22 credible economic models of the cost of single-payer financing in the United States, from a variety of government, business consultant, and academic organizations. We found that 19 (86%) predict net savings in the first year of operations, with a range of 7% higher net cost to 15% lower net cost. Increases in cost due to improved insurance coverage and thus higher utilization were 2% to 19%. Savings from simplified payment administration at insurers and providers, drug cost reductions, and other mechanisms ranged from 3% to 27%. The largest net savings were for plans with reductions in drug costs. Net savings accumulate over time at an estimated 1.4% per year. Of note, we excluded two widely publicized studies (Urban Institute 2016, Thorpe 2016), which found net costs, for making assumptions that included private insurance intermediaries (i.e. not a single payer) and for lacking technical detail for evaluation. |
| Limitations | 25 | Discuss limitations at study and outcome level (e.g., risk of bias), and at review-level (e.g., incomplete retrieval of identified research, reporting bias). | Limitations | Entire Section: Limitations |
| Conclusions | 26 | Provide a general interpretation of the results in the context of other evidence, and implications for future research. | Policy Implications | Entire Section: Policy implications |
| FUNDING | | |  |  |
| Funding | 27 | Describe sources of funding for the systematic review and other support (e.g., supply of data); role of funders for the systematic review. | Financial disclosure | CC JR IO and KB received $750 each from  Physicians for a National Healthcare Program  For a summer research grant. PNHP had no role  In the design, analysis or publication of this data. |

***Table G.*** *PRISMA Checklist. From: Moher D, Liberati A, Tetzlaff J, Altman DG, The PRISMA Group (2009). Preferred Reporting Items for Systematic Reviews and Meta-Analyses: The PRISMA Statement. PLoS Med 6(7): e1000097. doi:10.1371/journal.pmed1000097.* For more information, visit: www.prisma-statement.org.

**Text A Long-Term Projections**

Ten of the included studies included data beyond year one of implementation. All studies project increased savings as time continues after single payer implementation. Thus, conservatively, all studies show net savings 10 years after implementation, with an average increase in net savings of 1.4% per year (see linear regression). Analysis details:

- Independent variable was years after single-payer implementation
- Dependent variable was % net changes to health spending
- Regression was calculated using the linear regression tool on Microsoft Excel

**Figure A. Long-Term % Net Changes to Total Health Spending after Single-Implementation**


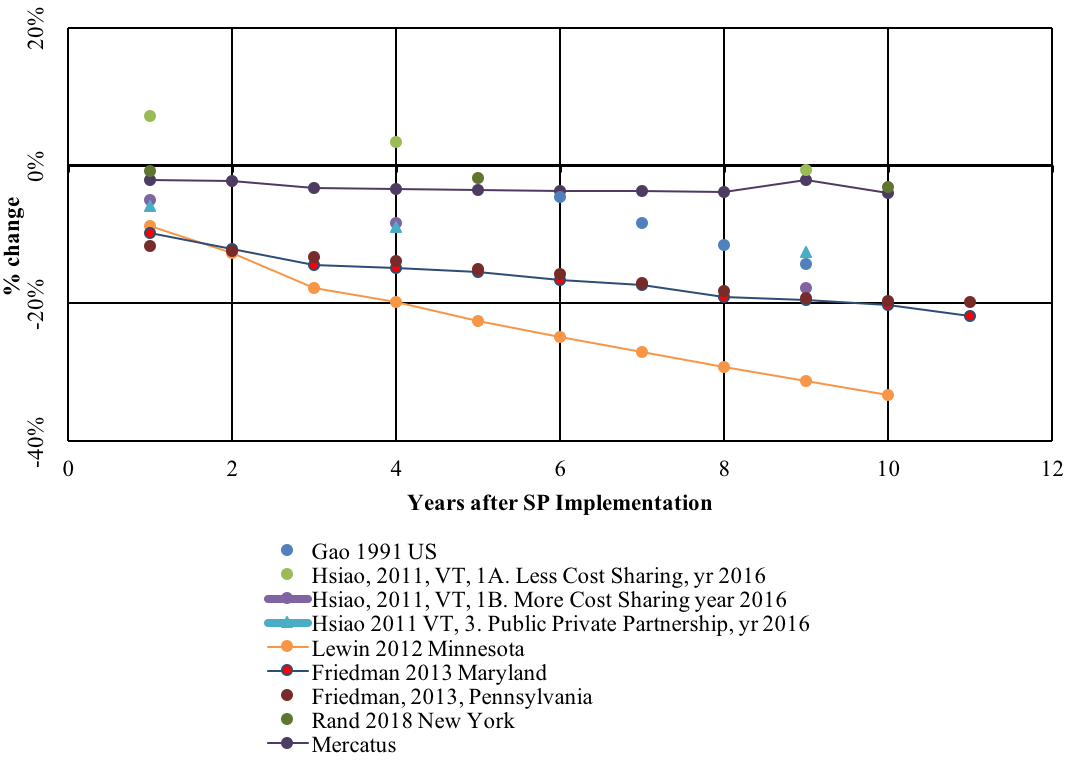


***Figure A****. Long-Term % Net Changes to Total Health Spending after Single-Implementation. “This figure shows the accumulation of net savings in annual health system costs over time, due to the use of global budgeting to control rate of growth.”*

**Figure B. Long-Term % Net Changes to Total Health Spending after Single-Implementation, with Trend-line**


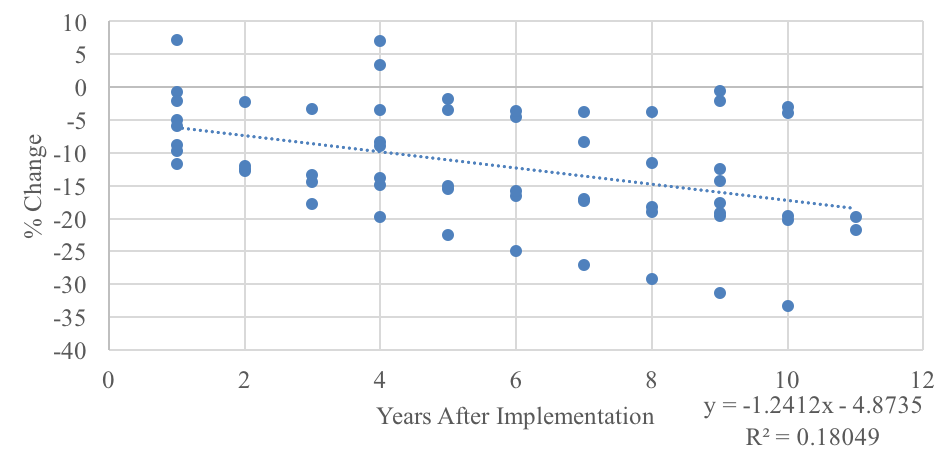


***Figure B*** *Long-Term % Net Changes to Total Health Spending after Single-Implementation, with Trend-line. This figure shows the same data as Figure A in S1 but with trend-line. Each dot represents one primary analysis. The dotted blue line represents a linear regression, with displayed results indicating the regression equation (including intercept and slope) and R-squared (proportion of variation explained).*

**Table H. Multivariate Regression Results**


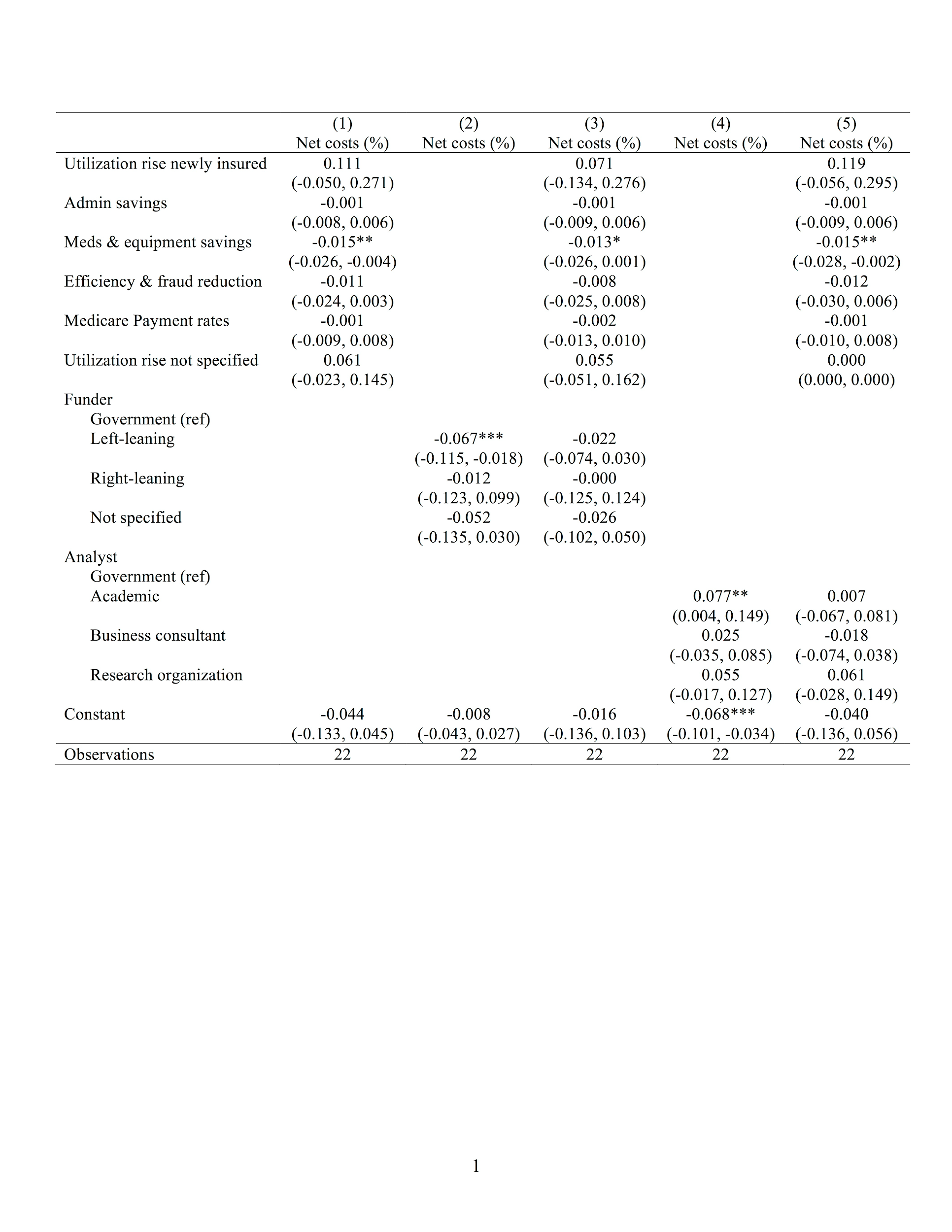


*Table H. Multivariable Regression Results. Significance: * p < 0.10 ** p < 0.05 *** p < 0.01*

**Table I. Regression input values.**

| Legend  Funder type: G = government, PL = politically left, PR = Politically right, N = not specified.  Analyst type: A = academic, B = business consultant, R = research organization, G = government.  Y is a dummy for non-zero unspecified value for utilization rise. |
| --- |

|  | Utilization rise newly insured (% of insured) | Admin savings | Meds / equip savings | Efficiency & fraud reduction | Medicare Payment rates | Funder type | Analyst type | **Net Costs** |
| --- | --- | --- | --- | --- | --- | --- | --- | --- |
| Hsiao, 2011, Vermont, 1A | 80.0% | 6.80 | 0.00 | 5.00 | 0.00 | G | A | 7.165% |
| CBO, 1993, U.S., SP2 | 50.2% | 6.95 | 0.00 | 0.00 | 0.00 | G | G | 5.025% |
| RAND, 2017, OR | Y | 1.83 | 0.00 | 0.00 | 10.00 | G | R | 0.296% |
| GAO, 1991, U.S. | 40.0% | 9.46 | 0.00 | 0.00 | 0.00 | G | G | -0.424% |
| RAND, 2018, NY | Y | 4.47 | 0.00 | 0.00 | 0.00 | PL | R | -0.739% |
| NHP, 1991, U.S. | 19.5% | 5.32 | 0.00 | 0.00 | 0.00 | PL | A | -1.329% |
| Lewin Group 2007 Maryland | 41.0% | 1.09 | 0.17 | 0.00 | 0.00 | PL | B | -1.663% |
| CBO, 1993, U.S., SP1 | 35.6% | 4.56 | 0.00 | 0.00 | 0.00 | G | G | -1.898% |
| Mercatus, 2018, U.S. | 45.0% | 1.82 | 1.34 | 0.00 | 8.42 | PR | A | -2.039% |
| Lewin, 2002, California CalCare | 41.0% | 9.29 | 2.64 | 0.00 | 0.00 | G | B | -2.437% |
| Liu 2016 More Generous | Y | 9.35 | 4.65 | 0.00 | 3.56 | N | R | -3.388% |
| Solutions for Progress, 2000, MA | 56.8% | 9.78 | 0.00 | 2.17 | 1.36 | PL | B | -3.533% |
| Hsiao, 2011, Vermont, 1B | 80.0% | 6.80 | 0.00 | 5.00 | 0.00 | G | A | -4.346% |
| Lewin, 2002, California, SPP | 41.0% | 8.70 | 2.64 | 0.00 | 0.00 | G | B | -5.007% |
| Hsiao, 2011, Vermont, 3 | 80.0% | 6.80 | 0.00 | 5.00 | 0.00 | G | A | -5.841% |
| Friedman, 2013, US | 25.0% | 15.10 | 3.70 | 0.00 | 0.00 | PL | A | -6.262% |
| Lewin, 2012, Minnesota | 41.0% | 10.19 | 1.89 | 0.44 | 0.00 | N | B | -8.751% |
| Pollin 2018, U.S. Sanders | 56.0% | 8.90 | 6.00 | 1.50 | 2.80 | PL | A | -9.666% |
| Pollin, 2017, California | 50.0% | 6.70 | 3.40 | 5.00 | 2.90 | PL | A | -10.105% |
| Friedman, 2013, Pennsylvania | 25.0% | 14.79 | 5.51 | 1.90 | 0.00 | PL | A | -11.697% |
| Friedman, 2013, Maryland | 45.0% | 16.39 | 7.92 | 0.00 | 0.00 | PL | A | -14.733% |
| Friedman 2015, NYHA | 30.0% | 17.12 | 5.67 | 1.88 | 0.00 | PL | A | -15.523% |

*Table I. Multivariable regression inputs. Made available for reproducibility.* This figure shows the input values used in regressions to predict net savings / costs, for each included analysis. The first 7 columns (all but the last) are right-side (predictor) variables. For Utilization, the value represents the increase due to becoming insured, as a % of costs for a fully insured person. The next four columns are types of savings, quantified as percent of health system costs. The next two columns are characteristics of the analysis. The final column is the left-side (outcome) variable, net costs (or, if negative value, savings). See main text for further description.
